# Endogenous and Exogenous Factors Affecting the Surgical Technique (Review)

**DOI:** 10.17691/stm2020.12.2.12

**Published:** 2020

**Authors:** A.E. Bykanov, D.I. Pitskhelauri, N.S. Grachev, D.E. Semenov, R.A. Sufianov, K.S. Yashin, K.B. Matuev

**Affiliations:** Researcher, N.N. Burdenko National Medical Research Center for Neurosurgery, Ministry of Health of the Russian Federation, 16, 4^th^ Tverskaya-Yamskaya St., Moscow, 125047, Russia;; Professor, Head of the 7^th^ Neurosurgical Department, N.N. Burdenko National Medical Research Center for Neurosurgery, Ministry of Health of the Russian Federation, 16, 4^th^ Tverskaya-Yamskaya St., Moscow, 125047, Russia;; PhD Student, N.N. Burdenko National Medical Research Center for Neurosurgery, Ministry of Health of the Russian Federation, 16, 4^th^ Tverskaya-Yamskaya St., Moscow, 125047, Russia;; Student, Faculty of Medicine, I.M. Sechenov First Moscow State Medical University (Sechenov University), 8/2 Malaya Trubetskaya St., Moscow, 119991, Russia;; Clinical Resident, 7^th^ Neurosurgical Department, N.N. Burdenko National Medical Research Center for Neurosurgery, Ministry of Health of the Russian Federation, 16, 4^th^ Tverskaya-Yamskaya St., Moscow, 125047, Russia;; Assistant, Department of Traumatology, Orthopedics, and Neurosurgery, Privolzhsky Research Medical University, 10/1 Minin and Pozharsky Square, Nizhny Novgorod, 603005, Russia;; Professor, Head of the Scientific and Educational Department, N.N. Burdenko National Medical Research Center for Neurosurgery, Ministry of Health of the Russian Federation, 16, 4^th^ Tverskaya-Yamskaya St., Moscow, 125047, Russia

**Keywords:** microsurgery, surgical technique, microsurgical training, precise movements in surgery

## Abstract

In this review, we analyzed essential factors affecting precise manual movements in microsurgery described in the medical literature. The search for publications in English and Russian languages was conducted in the PubMed database without limitation by the date of publication. The search was carried out according to the following descriptors: surgical procedures, dexterity, microsurgery, caffeine, alcohol, nicotine, physical exercise, sleep deprivation, posture. Only randomized and cohort studies involving doctors and students with surgical specialties were included in the analysis. We did not include papers in which only psychological (non-motor) aspects were studied.

Due to the limited number of publications meeting the inclusion criteria and conflicting results in some of them, the presented review does not allow us to formulate unambiguous conclusions and recommendations. Further studies (deep and fundamental) of endogenous and exogenous factors affecting the microsurgical technique are needed.

## Introduction

Continuous training and improvement of microsurgical technique throughout the professional career are mandatory for a qualified neurosurgeon. Often, developing high level microsurgical skills takes years of practicing [1–3].

Micro-neurosurgical technique is a combination of targeted and coordinated manual actions. Stability of a neurosurgical instrument in the hand of an operating surgeon is one of the most critical factors during surgery. First of all, it depends on the presence (or absence) of tremor, i.e., rhythmic hand movements resulting from involuntary contraction of agonists and antagonists muscles. This is especially relevant to microsurgery because hand movement of 1–2 mm caused by physiological tremor and not perceived otherwise, could cause problems when operating under a microscope using high magnification. Thus, the “firm” hand of the surgeon is one of the main factors affecting the outcome of neurosurgical operations, which is especially important in conditions of narrow and deep surgical wounds with minimally invasive neurosurgical approaches.

The pace and degree of microsurgical skill development differ between different young doctors.

## Exogenous factors

We use the term “exogenous” to define those external factors that are not genetically determined and that can be eliminated or modified. Among the most frequently discussed in the literature are the use of caffeine, alcohol, nicotine, the effect of physical activity, sleep disturbance and general fatigue, and the surgeon’s posture. Let us consider each of them in more detail.

***Caffeine.*** Caffeine consumption is common among health workers. According to studies [4, 5], 50 to 90% of medical personnel use caffeine in various forms (coffee, tea, energy drinks) during and after night shifts.

Most of the CNS effects of caffeine are due to its ability to bind and inhibit adenosine A1 and A2 receptors [6], which is based on the structural similarity between caffeine and adenosine molecules. Therefore, caffeine has a wide-range secondary effect on other receptors and, apparently, affects the transmission of dopamine, which plays a role in movement coordination [7].

Caffeine can enhance physiological resting tremor [8] — such with a frequency of 8–12 Hz is observed in almost all healthy people [4]. However, the dose of caffeine, enhancing resting tremor is rather individual and depends on the duration of exposure. In one study [9], it was shown that caffeine intake of more than 5 mg/kg of body weight significantly increased tremor; other authors [10] determined a threshold for a single dose caffeine negative effect as 300 mg.

Of studies on the effects of caffeine that met our criteria for inclusion in the review [8–16], 4 articles reported its negative effect on the surgical technique [8, 11–13], 2 articles reported no effect [14, 15], and only 1 study concluded that caffeine had a positive effect on surgical technique: specifically, it reduced the time taken to complete a task in sleep-deprived subjects [16] (see the Table).

**Table:** Influence of exogenous factors on surgical performance

Factors	Impact on surgical technique			Level of evidence
	Positive	Neutral	Negative	
Caffeine	Aggarwal et al., 2011 [16]	Pointdujour et al., 2011 [14]; Mürbe et al., 2001 [15]	Arnold et al., 1993 [8]; Humayun et al., 1997 [13]; Urso-Baiarda et al., 2007 [12]; Quan et al., 2015 [11]	Low
Alcohol			Dorafshar et al., 2002 [17]; Kocher et al., 2006 [18]; Gallagher et al., 2011 [19]	Low
Nicotine	We found no studies on the effect of nicotine on microsurgical skills that fit the criteria for inclusion in the review			Low
Physical exercise			Al Omran et al., 2016 [20]; Simon and Dare, 1965 [21]; Mürbe et al., 2001 [15]; Hsu and Cooley, 2003 [22]	Low
Sleep disorders and fatigue		Reznick and Folse, 1987 [23]; Deaconson et al., 1988 [24]; Jakubowicz et al., 2005 [25]; Uchal et al., 2005 [26]; Lehmann et al., 2010 [27]; Erie et al., 2011 [28]; Schlosser et al., 2012 [29]; Yi et al., 2013 [30]; Olasky et al., 2014 [31]; Eastridge et al., 2003 [32]; Veddeng et al., 2014 [33]	Mürbe et al., 2001 [15]; Taffinder et al., 1998 [34]; Grantcharov et al., 2001 [35]; Eastridge et al., 2003 [32]; Kocher H. et al., 2006 [18]; Ayalon and Friedman, 2008 [36]; Kahol et al., 2008 [37]; Leff et al., 2008 [38]; Kahol et al., 2011 [39]; Ganju et al., 2012 [40]; Basaran et al., 2015 [41]; Tsafrir Z. et al., 2015 [42]	Low
Surgeon’s posture	Arnold et al., 1993 [8]; Ohta and Kuroiwa, 2000 [43]; Csόkay et al., 2009 [44]; Goto et al., 2013 [45]			Low

***Alcohol.*** Alcohol consumption is not that common among health workers as caffeine use; among neurosurgeons, in our opinion, this habit is extremely rare. However, a number of studies report on quite a frequent occurrence of alcoholism among medical personnel in comparison with the general population [46, 47] and a greater addiction to alcohol among doctors of surgical specialties [48].

According to the literature, alcoholism occurs in 3.8% of Austrian doctors [49], in 18% of Belgian doctors [50], and in 32% of Spanish doctors [51]. Therefore, we included this factor in the present study.

We found only three papers that met our inclusion criteria [17–19]. These studies indicate a negative impact of alcohol consumption on surgical technique in the participated subjects. These tests were performed using laparoscopic simulators.

From the perspective of microsurgery, it would be interesting to study the effect of alcohol on physiological tremor, since a number of studies have shown a decrease in the hand physiological tremor under low doses of alcohol, which might be due to its central blocking effects [52, 53]. This effect is significantly less pronounced in individuals who rarely drink alcohol in everyday life [54]. Obviously, alcohol cannot be recommended as a means of reducing tremor, as it causes severe cognitive impairment and addiction. Unfortunately, we found no scientific publications on the effect of alcohol on the manual microsurgical technique, which, in our opinion, deserves further research.

It is also necessary to take into account the post-toxic state caused by alcohol abuse and its effect on the surgical technique. For example, it was determined that even insignificant alcohol consumption the day before the operation increased the number of errors as detected by a surgical simulator [19]. Meanwhile, another study showed that the night’s sleep following alcohol consumption completely eliminated the aftermath of recent excessive drinking [17]. The long-term effects of regular alcohol consumption on surgical skills also require further study.

***Nicotine.*** This factor has a stimulating (and, in high doses, inhibitory) effect on cholinergic transmission in both the peripheral and central nervous system (mainly inducing dopamine production) [55]. Studies show a significant increase in the amplitude of physiological tremor in smokers compared with non-smokers [56, 57], which can last for a long time [58]. We, however, found no studies on the effect of nicotine on microsurgical skills that fit our inclusion criteria.

***Physical exercise.*** In most people, physiological tremor increases after exercise, but after some time, it returns to its initial level. In particular, the effect of aerobic exercise on tremor cannot be detected in 2 h after training [20]. Among doctors — microsurgeons, there is a strong trend to avoid intensive physical exercises before the operation to rule out any increase in physiological tremor. At the same time, performing prolonged surgical interventions is impossible without having general and special endurance achieved by regular sports and exercise.

In the four publications [15, 20–22] that meet the criteria for inclusion in this review, the authors draw conclusions about a negative impact of physical exercises on surgical technique. Specifically, exercise could significantly increase the amplitude of physiological tremor in surgeons, regardless of their surgical experience [15]. Two studies analyzed the long-term effects of exercise on the tremors: a study of Mürbe et al. [15] showed that the increased amplitude of the tremor persisted for 24 h after the exercises, while in another study [22] — this effect lasted for only 4 h. A possible cause of this discrepancy may lie in different types of physical activity used in these two studies. Further work is needed to develop more accurate practical recommendations.

***Sleep disturbance and fatigue.*** Unfortunately, night shifts and sleep disturbances are an integral part of the doctor’s work, which may ultimately be the cause of psychological (mixed anxiety and depression) and physiological disorders [59, 60].

Fatigue and sleep deprivation can cause measurable biochemical changes in the blood and urine [61, 62]. Obviously, these factors do not favor the operating surgeon. However, the question is how critical are these factors for surgical performance and microsurgical technique? We analyzed a number of studies and reviews [12, 15, 18, 32, 34–38, 40–42] that showed a significant negative effect of sleep deprivation on surgical skills; in other works, on the contrary, no influence of this factor on surgical technique was found [23–33].

Study [38] showed that surgical skills were affected only during acute sleep deprivation (the first night shift), gradually recovering with an increase in the number of night shifts and a chronic lack of sleep. Thus, the inconsistent data does not allow us to draw unambiguous conclusions about the significance of this factor.

***The surgeon’s posture.*** Performing lengthy surgical interventions is often associated with the need to work in a standing position, with arms outstretched without having any support (see the Figure). Sometimes, a surgeon standing this way has to manipulate micro-tools up to 20 cm long. There are situations when it is technically possible to use a chair, armrests, various devices and the edges of a surgical wound as a hand-rest [43]. All quoted studies unanimously indicated a decrease in the tremor amplitude when using a hand rest [8, 44, 45, 63].

**Figure:**
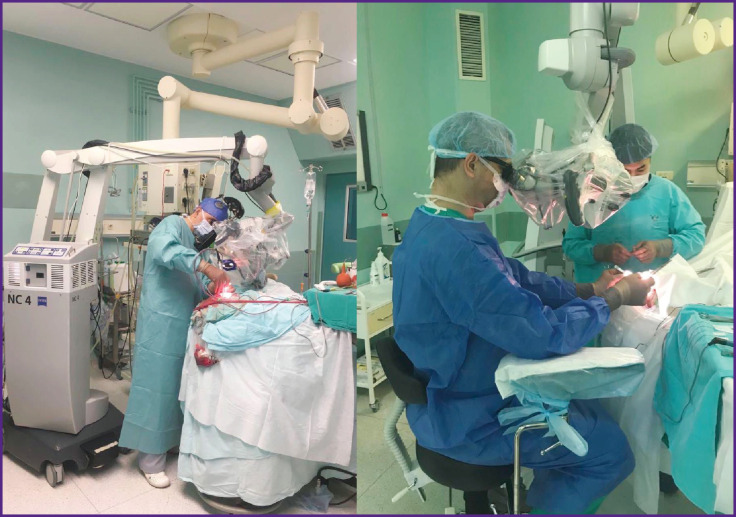
Possible options for the surgeon’s position (standing and sitting with a support for hands) during microsurgical operations

It is noted that some microsurgeons operate only while sitting and having support for their hands; other surgeons though prefer working in a standing position. We found no studies comparing surgical performance in a sitting and standing position.

## Endogenous factors

Concerning genetic differences between surgeons and their abilities to withstand prolonged physical loads, it should first be noted that human muscles are composed of slowly contracting (red) and rapidly contracting (white) muscle fibers [64]. These two types of fibers have different metabolic and morphological characteristics, and also contain different amount of crucial enzymes (creatine phosphokinase, phosphofructokinase, citrate synthase), with different activities in different fibers [65].

The ability of muscles to withstand physical loads (static or dynamic) depends on the relative content of different fibers. On average, the number of rapidly contracting fibers in human muscles comprises 55% of their total number. The presence of certain fibers in the muscles is a genetically determined factor that underlies the person’s predisposition to static or dynamic loads. Thus, slowly contracting fibers predominate in the muscles of marathon athletes. In contrast, sprint athletes largely have fast-contracting fibers [66, 67]. As for neurosurgery, this is, of course, a “marathon” profession, favorable for a neurosurgeon with predominantly slowly contracting muscle fibers.

It is worth noting that the number and distribution of fiber types can change with training [68–71]. The distribution of slowly and rapidly contracting fibers in the muscles of microsurgeons can significantly affect the rate of development and severity of fatigue during the operation, and, consequently, the severity of tremor. Numerous biologically active substances, signaling pathways, and their associated genes determine a specific phenotype of muscle fibers; these factors include MAPK, calcineurin, calcium/calmodulin-dependent protein kinase IV, and gamma coactivator of peroxisome-1 proliferation. Genes involved in the substitution of one type of fibers by another have been identified [72].

Historically, the differences in the metabolic activity of different muscles were studied by sports medicine in an attempt to reveal the changes caused by training and understand whether the structural differences were genetically determined. Using the methods of sports medicine for the selection or training of microsurgeons may become relevant one day.

## Conclusion

The ambiguity of the presented results suggests that exogenous factors have a minor influence on the development of microsurgical skills and “firm” hand of the microsurgeon. Therefore, future research should focus on endogenous factors, such as neuronal and muscular metabolic activity.

**Authors’ contribution:** A.E. Bykanov — study conceptualization and planning, drafting the manuscript; D.I. Pitskhelauri — final editing, scientific support; N.S. Grachev — selection and analysis of the literature; D.E. Semenov — data collecting and processing, writing, editing; R.A. Sufianov — selection and analysis of the literature; K.S. Yashin — selection and analysis of the literature; K.B. Matuev — text editing, scientific guidance.
